# Patterns of metabolic syndrome and associated factors in women from the ELSA-Brasil: a latent class analysis approach

**DOI:** 10.1590/0102-311XEN039923

**Published:** 2023-12-11

**Authors:** Nila Mara Smith Galvão, Sheila Maria Alvim de Matos, Maria da Conceição Chagas de Almeida, Ligia Gabrielli, Sandhi Maria Barreto, Estela M. L. Aquino, Maria Inês Schmidt, Leila Denise Alves Ferreira Amorim

**Affiliations:** 1 Universidade do Estado da Bahia, Salvador, Brasil.; 2 Instituto de Saúde Coletiva, Universidade Federal da Bahia, Salvador, Brasil.; 3 Instituto Gonçalo Moniz, Fundação Oswaldo Cruz, Salvador, Brasil.; 4 Faculdade de Medicina, Universidade Federal de Minas Gerais, Belo Horizonte, Brasil.; 5 Faculdade de Medicina, Universidade Federal do Rio Grande do Sul, Porto Alegre, Brasil.; 6 Instituto de Matemática e Estatística, Universidade Federal da Bahia, Salvador, Brasil.

**Keywords:** Metabolic Syndrome, Women, Latent Class Analysis, Social Determinants of Health, Síndrome Metabólica, Mulheres, Análise de Classes Latentes, Determinantes Sociais da Saúde, Síndrome Metabólico, Mujeres, Análisis de Clases Latentes, Determinantes Sociales de la Salud

## Abstract

This study aimed to identify patterns of metabolic syndrome among women and estimate their prevalence and relationship with sociodemographic and biological characteristics. In total, 5,836 women were evaluated using baseline data from the *Brazilian Longitudinal Study of Adult Health* (ELSA-Brasil). Patterns of metabolic syndrome were defined via latent class analysis, using the following metabolic abnormalities as indicators: abdominal obesity, hyperglycemia, hypertension, hypertriglyceridemia, and reduced HDL cholesterol. The relationship between these patterns and individual characteristics was assessed using latent class analysis with covariates. Three patterns of metabolic syndrome were identified: high metabolic expression, moderate metabolic expression, and low metabolic expression. The first two patterns represented most women (53.8%) in the study. Women with complete primary or secondary education and belonging to lower social classes were more likely to have higher metabolic expression. Black and mixed-race women were more likely to have moderate metabolic expression. Menopausal women aged 50 years and older were more often classified into patterns of greater health risk. This study addressed the heterogeneous nature of metabolic syndrome, identifying three distinct profiles for the syndrome among women. The combination of abdominal obesity, hyperglycemia, and hypertension represents the main metabolic profile found among ELSA-Brasil participants. Sociodemographic and biological factors were important predictors of patterns of metabolic syndrome.

## Resumo

O objetivo foi identificar padrões de síndrome metabólica em mulheres, estimar suas prevalências e relações com características sociodemográficas e biológicas. Este estudo examinou 5.836 mulheres utilizando dados da linha de base do *Estudo Longitudinal de Saúde do Adulto* (ELSA-Brasil). Os padrões de síndrome metabólica foram definidos por meio de análise de classe latente, usando as seguintes anormalidades metabólicas como indicadores: obesidade abdominal, hiperglicemia, hipertensão, hipertrigliceridemia e colesterol HDL reduzido. As relações entre os padrões de síndrome metabólica e as características individuais foram avaliadas por meio da análise de classes latentes com covariáveis. Foram identificados três padrões de síndrome metabólica, denominados “alta expressão metabólica”, “expressão metabólica moderada” e “baixa expressão metabólica”. Os dois primeiros padrões representaram a maioria (53,8%) das mulheres do estudo. As mulheres com nível de escolaridade primário ou secundário e pertencentes à classe social baixa tiveram maior chance de apresentar maior expressão metabólica. Negros e pardos tiveram maior chance de apresentar “expressão metabólica moderada”. Mulheres na menopausa com 50 anos ou mais apresentaram maior chance de ter padrões de maior risco à saúde. Este estudo abordou a natureza heterogênea da síndrome metabólica, identificando três perfis distintos para a síndrome entre as mulheres. A combinação de obesidade abdominal, hiperglicemia e hipertensão representa o principal perfil metabólico encontrado entre os participantes do ELSA-Brasil. Fatores sociodemográficos e biológicos foram importantes preditores para os padrões de síndrome metabólica.

## Resumen

El objetivo fue identificar patrones del síndrome metabólico en mujeres, estimar sus prevalencias y relaciones con características sociodemográficas y biológicas. Este estudio examinó 5.836 mujeres utilizando datos de la línea de base del *Estudio Longitudinal de Salud del Adulto* (ELSA-Brasil). Los patrones de síndrome metabólico se definieron a través del análisis de clase latente, utilizando las siguientes anormalidades metabólicas como indicadores: obesidad abdominal, hiperglucemia, hipertensión, hipertrigliceridemia y colesterol HDL reducido. Las relaciones entre los patrones de síndrome metabólico y las características individuales se evaluaron a través del análisis de clases latentes con covariables. Se identificaron tres patrones de síndrome metabólico, denominados “alta expresión metabólica”, “expresión metabólica moderada” y “baja expresión metabólica”. Los primeros dos patrones representan la mayoría (el 53,8%) de las mujeres del estudio. Las mujeres que tenían un nivel de escolaridad primario o secundario y que pertenecían a la clase social baja tuvieron una mayor probabilidad de presentar una expresión metabólica más alta. Los negros y pardos tuvieron una probabilidad más alta de presentar “expresión metabólica moderada”. Las mujeres en la menopausia que tenían 50 años o más presentaron una probabilidad más alta de tener patrones de mayor riesgo para la salud. Este estudio abordó la naturaleza heterogénea del síndrome metabólico, identificando tres perfiles diferentes para el síndrome entre las mujeres. La combinación de obesidad abdominal, hiperglucemia e hipertensión representa el principal perfil metabólico encontrado entre los participantes del ELSA-Brasil. Factores sociodemográficos y biológicos fueron importantes predictores para los patrones de síndrome metabólico.

## Introduction

Metabolic syndrome is a current and important public health problem worldwide ^1^ and consists of a set of interrelated metabolic abnormalities that occur simultaneously in the same individual ^2^ ^,^ ^3^. Over the past 20 years, different international health organizations have formalized several diagnostic criteria for metabolic syndrome ^3^. Although they differ in relation to the mandatory nature and cut-off points for some of the metabolic alterations that constitute the syndrome, contemporary diagnostic criteria converge around the dysfunctions for its identification: abdominal obesity, hyperglycemia, hypertension, low HDL cholesterol levels, and hypertriglyceridemia ^3^ ^,^ ^4^. The most current definition, proposed in 2009 based on a consensus between the International Diabetes Federation (IDF) and other international health organizations, established the presence of at least three of the five aforementioned conditions to reach a diagnosis of metabolic syndrome ^3^.

Metabolic syndrome affects about 1/4 of the world’s population and its prevalence may be even higher in specific regions and populations^2^ ^,^ ^5^ ^,^ ^6^. A study carried out in the United States, based on the*National Health and Nutrition Examination Survey* (NHANES, 2007-2012), found that 34.2% of all U.S. adults have been diagnosed with metabolic syndrome ^7^. In Brazil, an analysis using baseline data from the *Brazilian Longitudinal Study of Adult Health* (ELSA-Brasil, 2008-2010) also estimated the prevalence of metabolic syndrome at around 34% among individuals aged 35-74 years ^8^. Besides its high prevalence, metabolic syndrome is a premorbid condition ^9^ that predisposes individuals to an increased risk of type 2 diabetes mellitus and cardiovascular disease ^2^, which are important causes of morbidity and mortality in Brazil and in most countries worldwide, especially low- and middle-income countries ^6^ ^,^ ^10^ ^,^ ^11^. Moreover, metabolic syndrome has been associated with the development of diseases such as hepatic steatosis, depression, and some types of cancer, such as breast cancer ^1^ ^,^ ^12^ ^,^ ^13^.

Results of many studies have shown differences in the occurrence of metabolic syndrome by age and sex/gender. According to these studies, women, especially at older ages, are the most susceptible group to development of the syndrome and consequent diseases ^14^ ^,^ ^15^ ^,^ ^16^ ^,^ ^17^. A study using NHANES data from 2003 to 2012 showed a prevalence of metabolic syndrome of more than 50% among U.S. women over 60 years of age ^15^. Moreover, the findings of other studies have shown that an increasing number of young women have been affected by metabolic syndrome ^18^ ^,^ ^19^, which suggests the need for further research to clarify aspects of its occurrence among women.

Metabolic syndrome is a polymorphic disease with different clinical phenotypes, identified based on the various combinations of its components. These phenotypes can present different pathophysiology and consequences ^19^ ^,^ ^20^. They are also expected to discriminate between different at-risk populations, with potentially sex-specific distributions ^9^ ^,^ ^20^. While some authors have conducted separate analyses of the components of metabolic syndrome ^14^ ^,^ ^21^, other researchers have preferred to identify and describe its heterogeneity ^20^ ^,^ ^22^ ^,^ ^23^. A methodological approach recently incorporated into this latter type of analysis includes latent variable modeling, particularly latent class analysis (LCA) ^23^ ^,^ ^24^ ^,^ ^25^. The use of LCA can improve knowledge about the occurrence of metabolic syndrome by identifying and analyzing subtypes/patterns of the syndrome with relevant expressions in a specific population of interest.

To the best of our knowledge, no population-based study to investigate the occurrence of metabolic syndrome among women, addressing the heterogeneity of its expression, was conducted in Brazil, a middle-income country with high proportion of black and mixed-race individuals. Moreover, little is known about the heterogeneity of metabolic syndrome among women. Therefore, this study seeks to identify patterns of metabolic syndrome among ELSA-Brasil participants to estimate their prevalence and relationship with sociodemographic and biological characteristics.

## Methods

### Study design and population

This is an observational study, using the ELSA-Brasil baseline data (2008-2010), part of a multicenter cohort including 15,105 participants aged 35-74 years, both active and retired workers from Brazilian research institutions and universities, of which 8,218 were women. The main objective of the ELSA-Brasil is to investigate the incidence and progression of diabetes and cardiovascular diseases, considering social, psychological, occupational, environmental, behavioral, and biological factors ^26^ ^,^ ^27^. Aquino et al. ^26^ presents details on the ELSA-Brasil sampling process. Briefly, the sample was selected from 52,137 potential participants, based on the incidence of outcomes of interest in the Brazilian population, using a 5% significance level, an 80% statistical power, a 20% exposure prevalence, and a 2.0 relative risk.

Among ELSA-Brasil participants, women with complete data for the variables of interest were initially included in this study (n = 7,968). Asian and indigenous women were excluded from the analyses, as they represent specific ethnicities with a very low frequency in the eligible population (n = 306; 3.8%). Women in unnatural menopause or who had not menstruated for less than one year (perimenopause) (n = 1,714) and women with premature ovarian failure (who stopped menstruating before 40 years of age ^28^) (n = 112) were also excluded, as they potentially had a different metabolic profile to other women. Thus, the analyses included the baseline data for 5,836 women.

### Variables

The ELSA-Brasil involved the application of questionnaires, physical measurements, and examinations. A separate publication describes in detail the variables, procedures, and tools used in the study ^26^. This study describes the variables selected for its analyses.

#### Metabolic syndrome

The consensus definition of metabolic syndrome was used. It was proposed jointly by representatives of the IDF, the American Heart Association (AHA), and the U.S. National Heart, Lung, and Blood Institute (NHLBI) ^3^ and establishes the presence of three or more of the following metabolic conditions for the diagnosis of the syndrome (cut-off points for women): abdominal obesity (waist circumference ≥ 80cm); hyperglycemia (fasting glucose ≥ 100mg/dL); low levels of HDL cholesterol (HDL < 50mg/dL); hypertension (systolic blood pressure ≥ 130mmHg and/or diastolic blood pressure ≥ 85mmHg); and hypertriglyceridemia (triglycerides ≥ 150mg/dL). Regardless of the absence of metabolic abnormalities according to these cut-off points, the use of antihypertensive, hypolipidemic, or hypoglycemic medication and a previous diagnosis of diabetes are considered criteria to indicate the presence of the corresponding abnormalities. Binary indicators (presence/absence) for the five metabolic abnormalities were developed, defining the 32 possible combinations of responses to these indicators as phenotypes of metabolic syndrome.

#### Sociodemographic and biological factors

Age was categorized into four age groups (35-44, 45-54, 55-64, and 65-74 years). Ethnicity/skin color was self-reported: black, mixed-race, or white. Schooling levels were up to middle school (indicating the following answers: never attended school, incomplete/complete elementary/middle school or incomplete high school), high school (complete high school or incomplete undergraduate course), and college and higher (undergraduates and graduates from college/universities).

The social class variable is related to socio-occupational status and derived from a score based on the current occupation (or last occupation, for retirees), expected income (based on the schooling level), and income of the participants. This score was used to define seven categories of socio-occupational status or social classes, which were grouped into three levels: high (upper high and lower high); medium (upper medium, medium, and lower medium); and low (upper low and lower low). Andrade et al. ^29^ describes the details of the socio-occupational status variable, both the score and the categories.

Besides age, menopause is the main factor related to metabolic syndrome in women ^17^ ^,^ ^30^ ^,^ ^31^. This analysis only considers natural menopause, defined as the absence of menstruation after a period of one year or more, due to natural cessation of ovarian follicular function ^32^. The variable “menopause” includes two categories: no (women who still menstruate) and yes (postmenopausal women).

Due to the strong relationship between age and menopausal status, an age-menopause variable was defined by combining the categories of these two variables into four categories: (1) women up to 50 years of age who still menstruate; (2) menopausal women up to 50 years of age; (3) women over 50 years of age who still menstruate; and (4) menopausal women over 50 years of age. Age was dichotomized using the mean age at menopause among Brazilian women (50 years of age) ^33^.

### Data analysis

A descriptive analysis was conducted to characterize the women in this study. The prevalence of metabolic syndrome was estimated by the presence of at least three metabolic abnormalities, using the consensus definition ^3^.

Patterns of metabolic syndrome were identified based on the indicators of five metabolic abnormalities using LCA ^24^. These patterns correspond to mutually exclusive classes of women with similar profiles of metabolic syndrome expression, described by their prevalence and the probability of occurrence of each indicator, conditioned on the latent patterns (measurement model).

LCA models with two, three, and four latent classes were adjusted. To select the best model, the Akaike information criterion (AIC), the Bayesian information criterion (BIC) ^24^ ^,^ ^34^, and the Vuong-Lo-Mendell-Rubin likelihood ratio test, which compares the model with C classes and the model with C-1 classes ^34^ ^,^ ^35^, were analyzed together. Classification quality was measured using entropy ^24^ ^,^ ^34^. The interpretation of patterns of metabolic syndrome (classes) in each model was also considered when selecting the final model ^24^. For the LCA model to be valid, local independence must be met, that is, the indicators must be mutually independent, conditional on the latent classes ^24^ ^,^ ^36^. This requirement was verified by analyzing the standardized bivariate residuals of the final model ^37^, with a maximum percentage of 5% of absolute residuals greater than 1.96 being considered acceptable.

**Figure 1:**
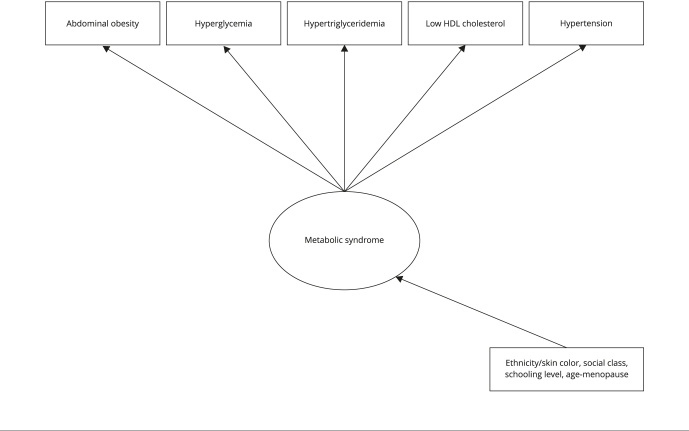
Latent classes analysis (LCA) model for metabolic syndrome among women participating in the *Brazilian Longitudinal Study of Adult Health* (ELSA-Brasil): indicators, latent variables, and associated factors.

LCA with covariates was used to assess the association between belonging to distinct patterns (classes) of metabolic syndrome and the covariates ethnicity/skin color, schooling level, social class, and age-menopause.Figure 1 shows the path diagram that represents the LCA model for metabolic syndrome, combining the measurement structure and the relationships with the covariates.

The measurement structure estimated from the final LCA model was the reference to assess the relationships between the metabolic syndrome construct and the covariates of interest, using LCA with covariates, which uses multinomial logistic regression. In a first phase, LCA with each of the covariates, separately, was adjusted. The invariance of the measurement model was assessed by comparing LCA with and without covariates ^24^. Finally, LCA with all covariates was adjusted. Crude and adjusted measures of association (crude and adjusted odds ratios and 95% confidence intervals) are presented.

Analyses were performed using Stata, version 12.0 (https://www.stata.com), and Mplus, version 8 (https://www.statmodel.com/).

### Ethical considerations

The ELSA-Brasil was approved by the Research Ethics Committee of each institution participating in the study and by the Brazilian National Research Ethics Committee ^26^ ^,^ ^27^. Participants signed an informed consent form at each phase of the cohort ^26^ ^,^ ^38^.

## Results

Table 1 presents the sociodemographic and biological characteristics of our study population. Women under 55 years of age (65.1%) and white participants predominated (55.8%). Most women (55.4%) had studied up to high school and belonged to the middle class (48.2%). About 45% (150 + 2,508 = 2,658) of women were postmenopausal and, among these, 5.6% (150/2,658) were under 50 years of age. In total, 83.6% had at least one of the components of metabolic syndrome, the most common being abdominal obesity or the combination of abdominal obesity and hyperglycemia. The prevalence of metabolic syndrome was 35.2% (presence of three or more metabolic abnormalities). Among black and mixed-race women and lower educated participants, this prevalence was over 40% (results not shown). Moreover, 381 women (6.5%) had all five components of the syndrome simultaneously.

**Table 1:** Characteristics of women participating in the *Brazilian Longitudinal Study of Adult Health* (ELSA-Brasil), 2008-2010.

Characteristics	n (5,836)	%
Age group (years)		
35-44	1,581	27.1
45-54	2,215	38.0
55-64	1,52	26.0
65-74	520	8.9
Ethnicity/Skin color		
Black	994	17.0
Mixed-race	1,586	27.2
White	3,256	55.8
Schooling level		
Up to middle school	520	8.9
High school	2,083	35.7
College and higher	3,233	55.4
Social class		
Low	1,199	20.5
Middle	2,811	48.2
Upper	1,826	31.3
Menopause		
No	3,178	55.4
Yes	2,658	45.5
Age-menopause (years)		
Up to 50 - no	2,704	46.3
Over 50 - no *	474	8.1
Up to 50 - yes **	150	2.6
Over 50 - yes	2,508	43.0
Number of metabolic disorders		
0	957	16.4
1	1,454	24.9
2	1,37	23.5
3	1,044	17.9
4	630	10.8
5	381	6.5

* The maximum age in this category is 68 years;

** All women in this category aged over 40 years.

We analyzed the 32 clinical phenotypes of metabolic syndrome using LCA to identify the smallest number of classes/subtypes of the syndrome with relevant expression for the women in the study and compared the measures of goodness of fit of the LCA models with two, three, and four latent classes (Table 2). The lower values of the AIC and BIC criteria and the results of the Vuong-Lo-Mendell-Rubin test showed that the four-class model was the best. However, the three-class model had improved entropy. The interpretability of the latent patterns led to the choice of the model with three latent classes, which met the assumption of local independence.

**Table 2:** Comparison of latent class analyses for metabolic syndrome with two, three, and four classes, according to model selection criteria.*Brazilian Longitudinal Study of Adult Health* (ELSA-Brasil), 2008-2010.

Classes	AIC	BIC	Entropy	VLMR	% SBR
2	33,974.6	34,047.9	0.62	0.000	55.0
3	33,593.9	33,707.4	0.65	0.000	2.5
4	33,489.4	33,642.8	0.59	0.000	0.0

AIC: Akaike information criterion; BIC: Bayesian information criterion; VLMR: Vuong-Lo-Mendell-Rubin likelihood ratio test (p-value); % SBR: proportion of standardized bivariate residuals > |1.96|.

Table 3 shows the estimates of LCA and the relative frequencies for each of the metabolic indicators. Overall, this study found a high prevalence of abdominal obesity (70%) and hyperglycemia (41%) and a significant frequency of hypertension (37%). We empirically identified three patterns of metabolic syndrome. The pattern characterized by high probabilities (> 0.5) for all five metabolic abnormalities was called “high metabolic expression” and involved 16.4% of women. The common occurrence of hypertriglyceridemia and reduced levels of HDL cholesterol clearly differentiate this pattern from the behavior observed in the overall population. The pattern mainly related to central obesity, hyperglycemia, and hypertension was called “moderate metabolic expression”, with a prevalence of 37.4%. The last pattern was characterized by low probabilities of presenting all abnormalities, except for central adiposity (47%), and was called “low metabolic expression”, including 46.2% of the women in this study.

**Table 3:** Characterization of latent patterns of metabolic syndrome via latent class analysis for women in the *Brazilian Longitudinal Study of Adult Health* (ELSA-Brasil), 2008-2010.

	Patterns of metabolic syndrome			
		High metabolic expression	Moderate metabolic expression	Low metabolic expression
Prevalence (%)		16.4	37.4	46.2
Indicators	Overall population	Conditional probabilities *		
Abdominal obesity	0.70	0.87	0.93	0.47
Hyperglycemia	0.41	0.67	0.65	0.16
Hypertriglyceridemia	0.25	1.00	0.26	0.04
Low HDL cholesterol	0.29	1.00	0.24	0.13
Hypertension	0.37	0.61	0.56	0.15

* Values in bold refer to prevalence rates at least 25% higher or 25% lower than those observed in the overall population.

Table 4 presents the estimated odds ratios (OR) between the three patterns of metabolic syndrome and the sociodemographic and biological variables. “Low metabolic expression” was the reference pattern. In the crude analysis, the following levels of the variables significantly increased the chance of higher metabolic expression compared with the reference: up to middle school (OR = 5.25; 95%CI: 3.56-7.75) and high school (OR = 1.63; 95%CI: 1.34-1.98); lower social class (OR = 2.44; 95%CI: 1.89-3.17); over 50 years of age without menopause (OR = 2.79; 95%CI: 1.86-4.18) and over 50 years of age in menopause (OR = 11.12; 95%CI: 8.67-14.26). In turn, moderate metabolic expression was positively associated with all covariates.

**Table 4:** Estimates of association between sociodemographic and biological characteristics and latent patterns of metabolic syndrome in women from the *Brazilian Longitudinal Study of Adult Health* (ELSA-Brasil), 2008-2010.

Covariates *	Patterns of metabolic syndrome **			
	High metabolic expression		Moderate metabolic expression	
	Crude OR (95%CI)	Adjusted OR (95%CI)	Crude OR (95%CI)	Adjusted OR (95%CI)
Ethnicity/Skin color				
Mixed-race	1.02 (0.82-1.25)	1.11 (0.88-1.40)	1.73 (1.43-2.09)	1.69 (1.35-2.11)
Black	1.03 (0.77-1.38)	1.21 (0.89-1.64)	2.73 (2.15-3.47)	2.73 (2.06-3.61)
Schooling level				
Up to middle school	5.25 (3.56-7.75)	-	8.26 (5.81-1.73)	-
High school	1.63 (1.34-1.98)	-	2.15 (1.79-2.59)	-
Social class				
Low	2.44 (1.89-3.17)	2.62 (1.97-3.48)	3.59 (2.81-4.60)	2.75 (2.09-3.61)
Middle	0.97 (0.79-1.19)	1.44 (1.14-1.81)	1.49 (1.22-1.82)	1.66 (1.32-2.07)
Age-Menopause (years)				
Over 50 - no	2.79 (1.86-4.18)	2.83(1.87-4.29)	4.68 (3.38-6.48)	5.04 (3.57-7.12)
Up to 50 - yes	1.72 (0.90-3.28)	1.57 (0.81-3.05)	2.05 (1.20-3.50)	1.84 (1.05-3.22)
Over 50 - yes	11.12 (8.67-14.26)	11.17 (8.61-14.50)	8.87 (6.97-11.29)	9.55 (7.45-12.23)

95%CI: 95% confidence interval; OR: odds ratio.

* Reference categories for covariates: white ethnicity/skin color, college and higher, higher social class, women aged 35-49 years who still menstruate;

** The “low metabolic expression” pattern was a reference class in latent classes analysis (LCA) with covariates.

Considering the strong relationship between schooling level and social class (χ^2^ = 48.21; p = 0.000), we included only one of them (social class) in the final LCA model. The simultaneous adjustment of the model for these covariates underestimates their association with latent patterns of metabolic syndrome and overestimates their standard errors due to collinearity (results not shown).

Including ethnicity/skin color, social class, and age-menopause in the LCA, the covariates black and mixed-race ethnicity/skin color and menopause in younger women (< 50 years of age) did not significantly change the chance of belonging to the “high metabolic expression” pattern. Moreover, all the characteristics analyzed were positively and significantly associated with moderate metabolic expression. For example, postmenopausal women aged 50 years and older had a 9.6 times higher chance of having moderate metabolic expression than low metabolic expression (OR = 9.55; 95%CI: 7.45-12.23) compared with women under 50 years of age who still menstruate, after controlling for other covariates. When we included schooling level instead of social class in our model, it showed similar results. Moreover, controlling for ethnicity/skin color and age-menopause, women who studied up to middle school had 3.4 (OR = 3.41; 95%CI: 2.20-5.29) and 3.92 (OR = 3.92; 95%CI: 2.58-5.97) times higher chances of having high and moderate metabolic expression, respectively, compared with women who completed high school (results not shown).

## Discussion

LCA showed three patterns of metabolic syndrome with relevant expressions among the women participating in the ELSA-Brasil: high metabolic expression, moderate metabolic expression, and low metabolic expression. The first two patterns included most women (53.8%) in the study. Sociodemographic variables such as ethnicity/skin color, schooling level, social class, and age-menopause proved to be important predictors of the patterns of metabolic syndrome, significantly changing the chance of an individual belonging to a profile with a higher health risk.

The most frequent clinical phenotypes of metabolic syndrome were abdominal obesity only (16%), abdominal obesity + hyperglycemia (7.8%), and abdominal obesity + hyperglycemia + hypertension (7.8%) (results not shown). In Brazil, only one study, conducted in rural communities in Minas Gerais State, sought results for the different combinations of the components of metabolic syndrome ^39^, showing that the combination of abdominal obesity + low HDL + hypertension was the most common profile in women. In contrast to hyperglycemia as the second most important metabolic phenotype among women in the ELSA-Brasil, the “low HDL” factor contributed greatly to the metabolic profile in a previous study ^39^. These results point to the importance of the interaction between the accumulation of visceral fat and changes in glucose metabolism in the development of metabolic risk in this population. More population-based studies with direct measures of blood glucose are needed to assess whether this evidence can be confirmed in Brazilian women.

In a study with U.S. adults participating in NHANES III (1988-1994), the combination of hypertriglyceridemia, low HDL, and abdominal obesity was the most frequent phenotype in women under 65 years of age, while the most predominant among women aged 65 years and older included all five components ^20^. The main differences in the patterns found in our study may be related, among other aspects, to ethnicity, lifestyle, social, and environmental specificities of each population ^5^ ^,^ ^9^. However, abdominal obesity was the most common indicator of metabolic syndrome in all populations, which confirms the evidence that, among women, the phenotypes or clusters of metabolic syndrome include abdominal obesity and at least two other metabolic abnormalities ^17^ ^,^ ^20^.

The three latent patterns of metabolic syndrome proved adequate to represent the heterogeneity of the expression of the syndrome in women from the ELSA-Brasil. These LCA patterns were consistent with the distribution of clinical phenotypes. No woman with low metabolic expression was diagnosed with metabolic syndrome (by the consensus definition), while 94.3% of the women with high metabolic expression received the diagnosis of metabolic syndrome. Moreover, 39.8% of the women in this latter pattern simultaneously had all five metabolic abnormalities (results not shown).

A multiethnic sample of 6,776 Americans aged 45-84 years from the *Multi-Ethnic Study of Atherosclerosis* (MESA) (2000-2012) showed similar results ^40^. After applying LCA, they found three latent classes/patterns of metabolic syndrome: non-metabolic syndrome (healthy status for all indicators); low risk (higher prevalences of abdominal obesity and hypertension); and metabolic syndrome (high prevalence for all indicators, except for hyperglycemia), affecting 42.7%, 27.4%, and 29.9% of the women in the study, respectively. Although we could not directly compare these results with ours, the latent structure estimated for the two populations was similar, the main difference being the contribution of hyperglycemia. This abnormality was prevalent in the two patterns of higher metabolic risk identified in our study and almost absent in the American study, consistent with the prevalence of hyperglycemia in both populations (ELSA-Brasil: 41%; MESA: 27.8%).

Our results may contribute to the current debate on the conceptualization and usefulness of metabolic syndrome ^9^ ^,^ ^23^. An important issue is related to the lack of knowledge about possible significant subtypes of metabolic syndrome, which may explain satisfactorily well the variability among individuals diagnosed with the syndrome ^9^ ^,^ ^41^. The findings of this study add evidence to the findings of Riahi et al. ^40^, Ahanchi et al. ^23^, and Ahanchi et al. ^25^ on the presence of an underlying, non-dichotomous factor that explains the interdependence of metabolic abnormalities related to metabolic syndrome.

Black and mixed-race women were more likely to have moderate metabolic expression than white women in our study, but we found no statistically significant relationship between ethnicity/skin color and high metabolic expression. In a rough comparison, but in the same direction as our results, Barbosa et al. ^42^ showed that being black was a risk factor for metabolic syndrome in women living in the city of Salvador (Bahia State, Brazil). However, the authors found no association between mixed-race ethnicity/skin color and the occurrence of the syndrome, while controlling for sociodemographic and behavioral variables. In a study with American-Hispanic and non-Hispanic individuals from NHANES, Moore et al. ^7^ pointed that, among non-Hispanics, from 2007 to 2012, black women had an increased chance of having metabolic syndrome compared with white women (OR = 1.20; 95%CI: 1.02-1.40).

In general, black individuals, and particularly black women, are more likely to have hypertension, obesity, and type 2 diabetes ^43^ and less likely to have reduced HDL cholesterol and hypertriglyceridemia ^42^ ^,^ ^44^ than mixed-race or white individuals. In this study, as well as in the two aforementioned studies, the prevalence of hypertension, abdominal obesity, and hyperglycemia was higher in black women, with a decreasing gradient between mixed-race and white women, which is consistent with the magnitudes of the association estimates found for the high and moderate metabolic expression patterns.

Several studies have addressed the association of lower schooling levels and unfavorable socioeconomic status with the occurrence of the metabolic syndrome in women ^45^ ^,^ ^46^ ^,^ ^47^. In this study, lower schooling levels and social class were considered risk factors for the syndrome, associated with patterns of high and moderate metabolic expression, after controlling for other covariates. Since abdominal obesity, alterations in glucose metabolism, and hypertension were important factors in the composition of the syndrome for the population in this study, these findings probably accompany the inverse relationship between the prevalence of these metabolic abnormalities and schooling and socioeconomic levels in Brazilian women ^46^ ^,^ ^48^. Lower schooling levels and unfavorable social status shape living conditions and influence behaviors that can favor the development of metabolic syndrome ^49^.

The literature is well established that aging and menopause are risk factors for metabolic syndrome in women ^19^ ^,^ ^30^ ^,^ ^50^ ^,^ ^51^. Our study corroborates this by showing that the other categories (by age-menopause) had higher chances of belonging to the “high” and “moderate metabolic expression” patterns compared with non-menopausal younger women (reference level), highlighting their influence on the development of subtypes of metabolic syndrome. Older women, regardless of menopausal status, were more likely to belong to patterns of higher metabolic expression. High metabolic expression and moderate metabolic expression were more strongly associated with older women who still menstruate than with the younger menopausal women. These results suggest that postmenopausal women, regardless of age, have a higher chance of belonging to patterns of higher metabolic risk. Moreover, in this study, age 50 years and older was the main factor associated with the occurrence of two subtypes of metabolic syndrome, and the menopausal condition potentiates its effect.

In agreement with the results of this study, Figueiredo Neto et al. ^52^, in a cross-sectional study with women with climacteric symptoms (40-65 years of age), identified age as the most important risk factor for the onset of metabolic syndrome. These authors stated that menopause was not a risk factor for the syndrome when other sociodemographic characteristics, including age, were considered in the analysis. Unlike our study, their study did not include women in the reproductive cycle, as the menopausal transition phase (perimenopause) was used as a comparison group. In fact, the literature provides consistent evidence of an increased metabolic risk in perimenopausal and postmenopausal women compared with premenopausal women, regardless of age ^30^ ^,^ ^53^ ^,^ ^54^. However, the influence of the interaction between aging and menopause is still unclear ^55^. Thus, further research, particularly longitudinal, may increase the understanding of these relationships.

To assess the factors associated with metabolic syndrome, the analyses conducted in previous studies defined the syndrome as a dichotomous trait (presence/absence), using criteria proposed in the literature, and assessed its relationships with the risk factors using traditional regression models for binary outcomes. Thus, comparisons between other studies and ours should be made carefully. The estimates obtained using LCA with covariates were consistent with the expected relationships between the covariates and metabolic syndrome, suggesting the validity of the latent structure. Moreover, some relationships that could be hidden in the approach that considers the syndrome as a dichotomous observed trait (data not shown) were statistically significant, such as the one between mixed-race ethnicity/skin color and moderate metabolic expression. This is the first population-based study to consider the heterogeneity of the expression of metabolic syndrome, identifying distinct patterns among Brazilian women. For this purpose, we used a powerful and widely known latent variable modeling technique, which enabled the assessment of the relationship between relevant combinations of the metabolic components of the syndrome and covariates, which would not be feasible for the analysis of all phenotypes.

Despite the usefulness of the method and the criteria used to select the number of latent patterns/classes defined by LCA, the choice of the ideal number of classes and their interpretation is subjective, which can lead to different structures. However, the latent structure in this study proved to be coherent with the distribution of the indicators of metabolic syndrome for the study population. Moreover, the associations estimated in our study were consistent with other studies in the literature and with the use of other methodological approaches, such as multinomial logistic regression.

Although the study population was not representative of adult women at national level, especially rural residents, women under 35 years of age, and women in unemployment or extreme poverty, the large sample size and the social, racial, and regional diversity represented by the women included in this study enabled the assessment of the relationships between the factors of interest and metabolic syndrome, thus supporting the internal validity of the study. Although our results should not be generalized to the general population of women, our findings shed light on relationships that may be valid for population subgroups with characteristics similar to the women in this study.

One of the strengths of this study is the use of direct measures for all metabolic indicators of the syndrome. Despite this, the prevalence reported for some metabolic disorders, especially abdominal obesity, and consequently the prevalence of metabolic syndrome may be overestimated due to the cut-off points used to dichotomize this indicator ^56^. For our analyses, we defined abdominal obesity by adopting the cut-off point for waist circumference guided by the consensus criterion for women of ethnic origin in Central and South America (≥ 80cm) ^3^. Although this cut-off point is conservative compared with other criteria for the definition of metabolic syndrome, it is closer to the criteria suggested by Eickemberg et al. ^57^ for the diagnosis of abdominal obesity in white and mixed-race women among ELSA-Brasil participants. Knowledge of the predominance of white/mixed-race women in this study, together with corroboration of the results of other studies, support this choice.

Finally, the methodology adopted in this study, which involved the heterogeneous aspect of metabolic syndrome, allowed the identification of subgroups of women with different metabolic risk profiles and relationships that could be hidden in a conventional analysis of this syndrome. The combination of abdominal obesity, hyperglycemia, and hypertension represents the main metabolic profile found among ELSA-Brasil participants. Sociodemographic and biological factors were important predictors of patterns of metabolic syndrome. This knowledge broadens the understanding of the syndrome in women and can guide effective public policies aimed at preventing and reducing metabolic syndrome in women.
